# Rearrangeable and exchangeable optical module with system-on-chip for wearable functional near-infrared spectroscopy system

**DOI:** 10.1117/1.NPh.5.1.011007

**Published:** 2017-09-09

**Authors:** Tsukasa Funane, Takashi Numata, Hiroki Sato, Shinsuke Hiraizumi, Yuichi Hasegawa, Hidenobu Kuwabara, Kiyoshi Hasegawa, Masashi Kiguchi

**Affiliations:** a Hitachi, Ltd., Research & Development Group, Center for Exploratory Research, Hatoyama, Saitama, Japan; b Hitachi Kokusai Yagi Solutions Inc., Kodaira, Tokyo, Japan; c Hitachi High-Technologies Corporation, Minato-ku, Tokyo, Japan

**Keywords:** functional near-infrared spectroscopy, system-on-chip, module-based system, wearable

## Abstract

We developed a system-on-chip (SoC)-incorporated light-emitting diode (LED) and avalanche photodiode (APD) modules to improve the usability and flexibility of a fiberless wearable functional near-infrared spectroscopy (fNIRS) system. The SoC has a microprocessing unit and programmable circuits. The time division method and the lock-in method were used for separately detecting signals from different positions and signals of different wavelengths, respectively. Each module autonomously works for this time-divided-lock-in measurement with a high sensitivity for haired regions. By supplying +3.3  V of power and base and data clocks, the LED module emits both 730- and 855-nm wavelengths of light, amplitudes of which are modulated in each lock-in frequency generated from the base clock, and the APD module provides the lock-in detected signals synchronizing with the data clock. The SoC provided many functions, including automatic-power-control of the LED, automatic judgment of detected power level, and automatic-gain-control of the programmable gain amplifier. The number and the arrangement of modules can be adaptively changed by connecting this exchangeable modules in a daisy chain and setting the parameters dependent on the probing position. Therefore, users can configure a variety of arrangements (single- or multidistance combinations) of them with this module-based system.

## 1 Introduction

Functional near-infrared spectroscopy (fNIRS) measures the changes in cerebral hemodynamics and oxygenation by irradiating the head (scalp) with weak visible or near-infrared light and detecting the reflected (scattered) light from another position.^1^^–^^5^ The fNIRS technique has been applied to noninvasively obtain two-dimensional topographic or three-dimensional tomographic images (or diffuse optical tomographic images) of the brain hemodynamics and oxygenation changes responding to various cognitive tasks or during resting state.^6^^,^^7^ The fNIRS systems are being used more and more widely around the world,^8^ such as for neuroimaging research,^9^^,^^10^ medical purposes,^11^^–^^14^ and especially, for measuring the brain activity of infants and children,^15^^–^^21^ because they have a high level of safety^22^^,^^23^ and require few constraints.

Wearable (or portable wireless) fNIRS systems^24^^–^^33^ are becoming more and more important, particularly for social neuroscience studies for investigating teacher–student interaction,^34^^,^^35^ face-to-face communication, and real-world cognitive tasks.^36^ They are also being applied to oximeters,^37^ child brain research,^38^ and clinical purposes, such as mental health care.^39^^–^^41^ Such wearable, small systems are used in a hyperscanning configuration,^25^^,^^35^ where two or more brains are simultaneously measured and combined with other modalities, such as electroencephalogram.^42^ It has been demonstrated that mood states can be objectively measured^43^ especially in back-to-work programs^39^^,^^41^ and face-to-face interaction behaviors.^40^ Since those measurements are supposed to be performed in homes or offices, small and low-cost equipment, such as a wearable fNIRS system, is needed.

Some limitations of most fNIRS systems are, however, that optical probes (or optodes) are hard to replace, and that configurations and setting parameters of them are inflexible. Disorder in only one probe makes the system incomplete, and the probe is difficult for users to replace. The new concept shown in this paper solves these limitations. The concept consists of replaceable-optode [light-emitting diode/avalanche photodiode (LED/APD)] probe modules that have system-on-chip (SoC) with an embedded microprocessing unit (MPU) and flexible settings for a number of time divisions and frequencies that optimize the signal-to-noise ratio for a specific probe arrangement. The amount of peripheral circuits has been decreased by using the SoC that can dynamically and programmably configure both analog and digital components, which can save on component cost and reduce the size of modules. A module-based system can save on the maintenance and repair costs because modules can be easily replaced when one becomes out of order.

## 2 System Concept

**Fig. 1:**
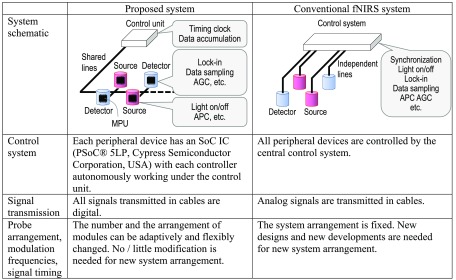
Conceptual comparison between proposed and conventional fNIRS systems.

In the proposed fNIRS system, each peripheral device (LED or APD module) has its controller (MPU), whereas in a conventional fNIRS system, all the sources and detectors are controlled by a central controller. Figure 1 shows a conceptual comparison between the proposed and conventional fNIRS systems.

In the conventional system, all peripheral devices are controlled by the central controller. Analog signals are transmitted in cables. The system arrangement (modulation frequency, light emitting timing, combination of sources and detectors) is fixed, so new designs and new developments are needed for new system arrangements. On the other hand, in the proposed system, each peripheral device has an SoC IC (PSoC® 5LP, Cypress Semiconductor Corporation, USA) with each MPU, which autonomously works under the control unit. All signals transmitted in cables are digital. The system has thus better noise characteristics. The number of modules, the arrangement of modules, modulation frequencies in lock-in detection, and number of time divisions can be adaptively and flexibly changed with commands from the control unit. Therefore, new system arrangements require little or no modification. This module-based system is suitable for a wearable and fiberless fNIRS system.

Table 1 summarizes module specifications. For practical equipment and low electricity consumption, power supply voltage is +3.3  V. The light guide diameter is ϕ3  mm for the approaching scalp for both LED and APD modules. For the LED module, wavelengths of light sources are 730 and 855 nm, and optical power output is 60 mWpp max. It has an automatic power control (APC) function. The modulation of an optical signal is a square wave with a 50% duty ratio and time-shared by one to four modules. As for the APD module, the detector is Si-APD (sensor diameter: 1.0 mm), and a time divided lock-in is used for signal decoding. Transimpedance amplifier (TIA) and automatic gain control (AGC) using a programmable gain amplifier (PGA) are incorporated. The sampling rate is 10  S/s. Each module is embedded in the system by connecting with a small connector to share a limited number of pins, such as power supplies, communication line (interintegrated circuit: $I^{2}C$), and clocks.

**Table 1:** Module specifications.

Power supply	+3.3 V
Light guide	ϕ3 for approaching scalp
Dimension	Width 22 mm, depth: 25 mm, height: 42 mm
**LED module**	
Light sources	730 and 855 nm LEDs
Optical power output	60 mWpp max, automatic power/current control
Modulation	Duty 50%, time division: 1 to 4
Power consumption	0.32 W (typical)
Weight	12.5 g
**APD module**	
Detector	Si-APD
Decoding	Time divided lock-in
Amplifier	Transimpedance amp, AGC using programmable gain amp
PGA range (i.e., dynamic range)	1 to 2450
Sampling rate	10 S/s
Power consumption	0.48 W (typical)
NEP (system)	2.7 pW (730 nm), 4.9 pW (855 nm)
Weight	9.7 g

**Fig. 2:**
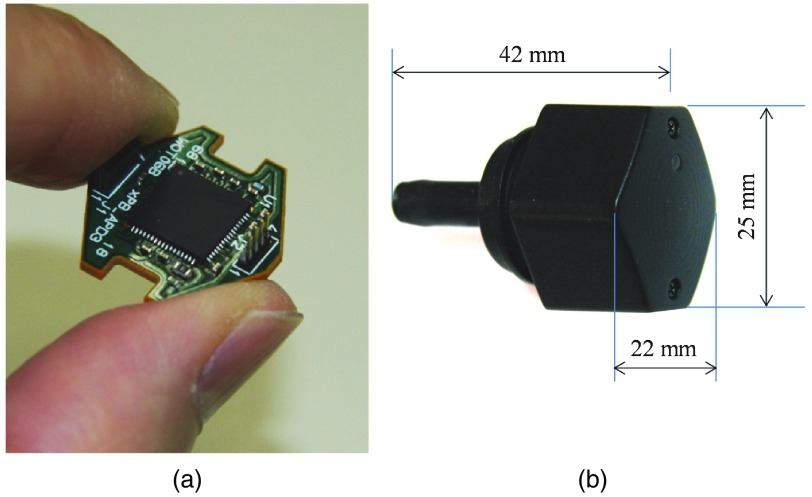
Photographs of (a) an electronic circuit board of APD module and (b) a case of LED/APD module.

Photographs of an electronic circuit board of an APD module and a case of LED/APD modules are shown in Fig. 2. The circuit size is determined by the requirement for probe arrangement: a source–detector (S-D) distance of around 30 mm.

**Fig. 3:**
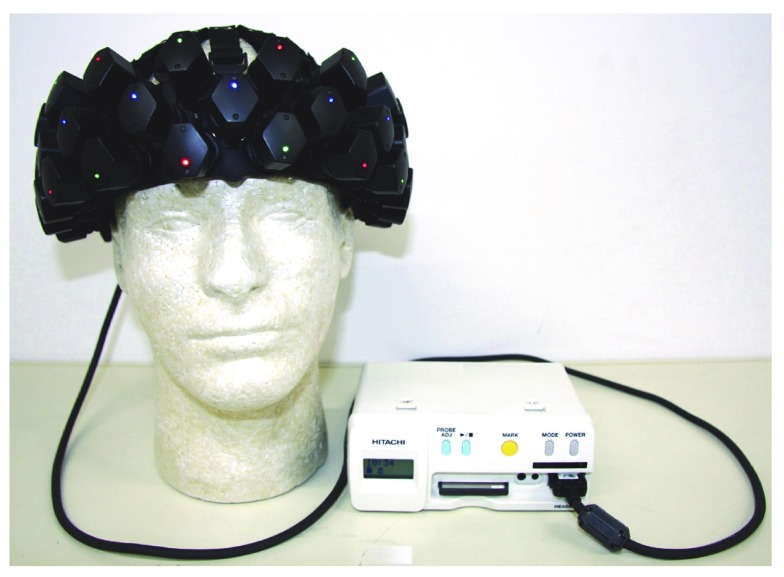
Photograph of wearable fNIRS system using LED and APD modules.

A photograph of a wearable fNIRS system using LED and APD modules is shown in Fig. 3. This system has 12 LED and 23 APD modules, and the total weight of modules and a plastic module holder (headset) is about 497 g. A flexible printed circuit (with small connectors) electrically connects among the modules. All the modules and the flexible printed circuit are supported by the holder with rubber parts. The control unit is inside a portable box.

**Fig. 4:**
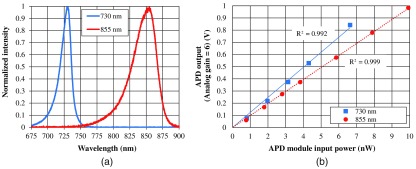
(a) Spectra of LED outputs. (b) Linearity between APD module input power and mean APD signal output after analog lock-in detection.

Spectra of LED outputs and the linearity between APD module input power and mean APD signal output after analog lock-in detection are shown in Fig. 4. Typical peak wavelengths for LEDs are 730 and 855 nm. Typical full-width at half-maximum values of output spectra are 20 and 30 nm for 730 and 855-nm LEDs, respectively.

## 3 Technology to Realize the Concept

### 3.1 Electrical Connection Among Modules

**Fig. 5:**
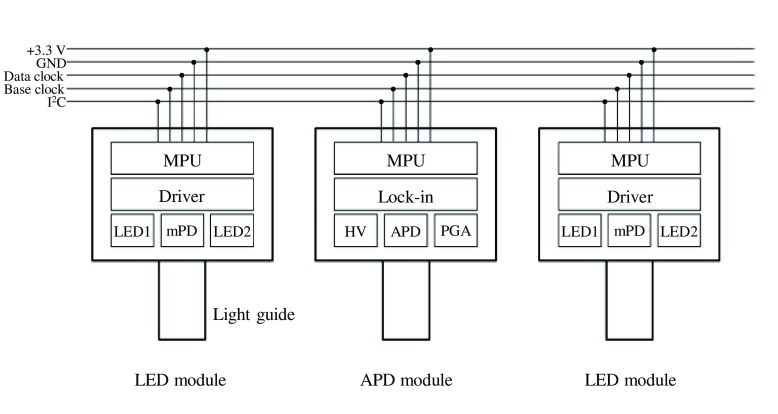
Schematic of optical modules. All analog circuits are built-in. MPU and HV represent microprocessing unit and high voltage supplier, respectively.

MPU and programmable analog/digital circuits can reconstruct the function of the system. Command-based operation of each module is realized. Optical modules are schematically shown in Fig. 5. All analog circuits are built-in. HV represent a high-voltage supplier. These optical modules are connected via an $I^{2}C$ bus, a daisy-chain communication protocol. Since the base and data clocks are shared, optical emission and detection timings are synchronized. Power supply for LED light sources, power supply for electronic circuits, ground, data clock, base clock (BC), and $I^{2}C$ bus (serial clock line and serial data line) are shared among all modules.

### 3.2 Basic Structure of LED/APD Module and Control Unit

**Fig. 6:**
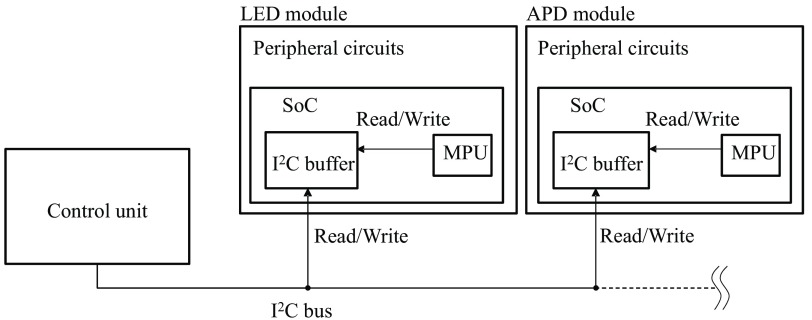
Schematic of communication network connections between LED/APD modules and control unit. Control unit plays a role of a master and SoCs play roles of slaves in the $I^{2}C$ bus.

LED/APD modules and control unit are connected to each other in a daisy-chain configuration using an $I^{2}C$ bus. Up to 128 slave addresses (i.e., modules) can be assigned in our system. These modules can be adequately applied to many kinds of measurements from one-channel to multichannel brain-activity monitoring. They share information using a memory buffer in each LED/APD module that is read or written by the control unit. The memory buffer includes setting parameters and measurement data that are read and written by an MPU. The control unit sends data to a personal computer (PC) via wired/wireless local area network and hemoglobin (Hb) calculation is performed by software at the PC. The molar extinction coefficients (as a function of wavelength) can be set for spectroscopy analysis (i.e., modified Beer–Lambert law^44^), and analog-to-digital (A/D) converted optical intensity signal at each light source can be saved in a text file as well. Figure 6 schematically shows the communication network connections between LED/APD modules and a control unit.

### 3.3 Frequency Generation

LED and APD modules generate frequencies for modulating/detecting optical signals by dividing the BC, 163,840 Hz, from the control unit. The number of divisions can be set to any integer value. By using programmable frequency divider components and flip-flop circuits, over six frequencies of a common divisor can be generated. The frequency phases are easy to synchronize because the frequencies share the same BC.

**Fig. 7:**
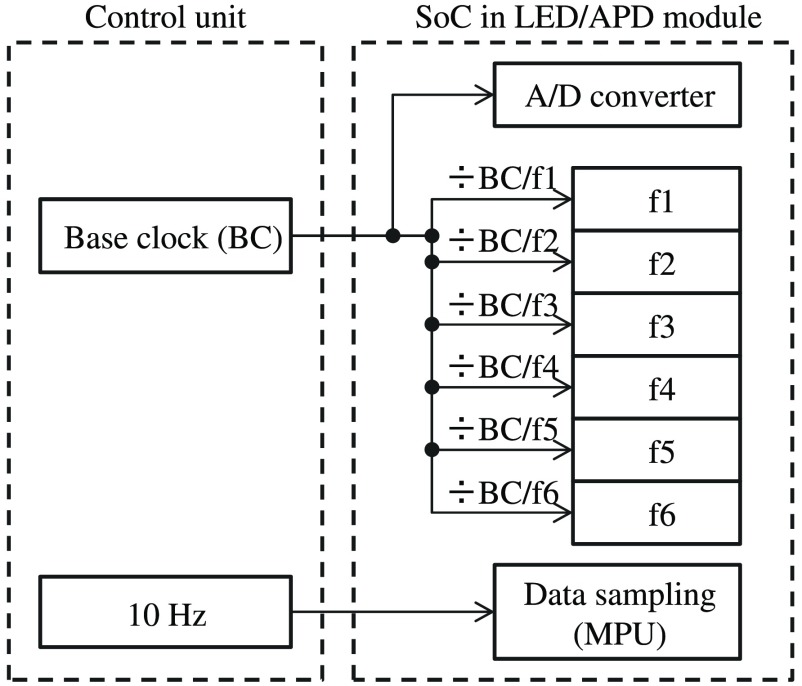
Generation of clocks at LED/APD modules for A/D conversion, frequency modulation, and data sampling.

A schematic of generation of clocks at LED/APD modules for analog-to-digital (A/D) conversion (ADC), frequency modulation, and data sampling is shown in Fig. 7.

### 3.4 Time-Division and Frequency-Division Lock-in Detection

Up to four positions of light sources are discriminated by timing of optical signals, and two wavelengths are discriminated by two frequencies of modulation. Frequencies of the first and second nearest sources emitted at the same time are set differently to avoid crosstalk. Since each APD module can detect two signals (i.e., wavelengths) at the same time, more than two signals should be separately detected by time divisions (details are stated in Sec. 3.7). Table 2 shows a relationship between probe arrangement and conditions for time-division lock-in detection [minimum number of time divisions (T) and number of frequency divisions (F)]. The probe arrangements are assumed as 30-mm lattice arrangements with light source on top left position. In all probe arrangements, minimum numbers of frequency divisions (F) are two, with reference to a practical criteria mentioned in Sec. 3.5, but they can be set to more than two with the same or better signal quality.

**Table 2:** Relationship between probe arrangement and conditions for time-division lock-in detection.

Probe arrangement (30-mm lattice arrangement with light source on top left)	Minimum number of time divisions (T)	Minimum number of frequency divisions (F)
1×2	1	2
1×N (N≧3)	2	2
2×2	2	2
2×N (N≧3)	3	2
3×3	3	2
M×N (M≧3, N≧4)	4	2

Atsumori et al.^24^ reported time-divided digital lock-in detection, which enables cross-talk-free measurements because no multiple light sources are simultaneously emitted. In this technology, multiple lights modulated in different frequencies are simultaneously irradiated on the scalp to detect and separate the multiple lights emitted from different light sources (frequency lock-in detection method). Time-divided digital lock-in detection is useful for biological optical measurements, such as simultaneous multiple-point measurements, but these frequencies must be set sufficiently far apart or the light sources should be placed sufficiently far apart to prevent interference between light sources.

### 3.5 Possible Probe Arrangements

**Fig. 8:**
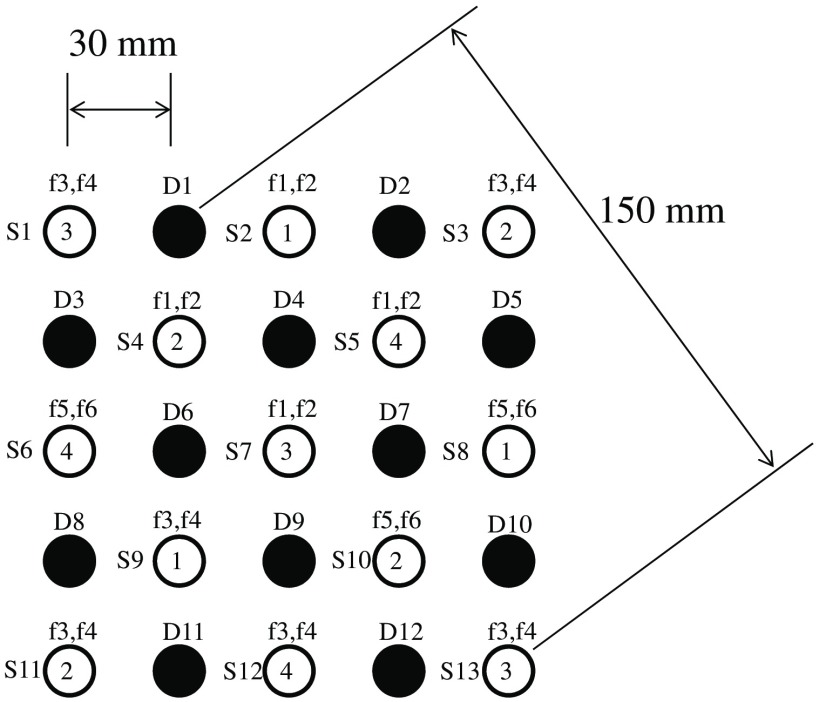
Example 5×5 probe arrangement using 4 time divisions and 6 frequencies. White and black circles indicate positions for light sources (LEDs, S1–13) and detectors (APDs, D1–12). The numbers in a white circle indicate light-emitting timings. The numbers with “f” above light sources are frequency indices.

An example 5×5 probe arrangement using four time divisions and six frequencies is shown in Fig. 8. Ideally, there should be as many frequencies as light sources used simultaneously, but the same frequencies can be used simultaneously at distant positions from each other. In the case of Fig. 8, detector No. 1 (D1) detects signals of frequencies 3 and 4 (f3 and f4) at division 3 (number in light-source circle) from both sources No. 1 (S1) and No. 13 (S13). However, S-D distances for S1-D1 and S13-D1 combinations are 30 and 150 mm, respectively, and the latter signal is much weaker than the former one (which can be considered to be zero).

**Fig. 9:**
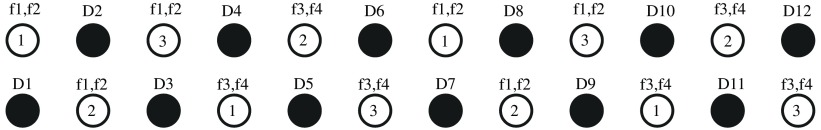
Example 2×12 probe arrangement using 3 time divisions and 4 frequencies. White and black circles indicate positions for light sources (LEDs) and detectors (APDs), respectively.

When frequencies and timing can be changed individually, a 2×12 probe arrangement using only three time divisions and four frequencies, for example, can be configured, as shown in Fig. 9.

The rearrangeable concept is useful for optimal measurements depending on the purpose (signal-to-noise ratio, number of measurement positions, etc.). The distance between LED and APD modules is preferably set to 30 mm because it is reported that a cerebral signal can be detected on a human scalp with sources and detectors 30 mm apart.^45^

When the distance between LED and APD modules on a human scalp is expanded from 30 to 50 mm, the detected optical intensity exponentially reduces to one-hundredth (1%).^46^ In other words, the intensity reduces to one-tenth at each 10 mm. For one APD module, the intensity of the signal from an LED module 65 mm away is less than one-thousandth of that from an LED module 30 mm away, which can be practically ignored. In this way, for one APD, there should be no more than one LED module modulated with the same frequency emitting at the same time within a 65 mm distance. Therefore, any 30-mm lattice arrangement can be configured by using only four time divisions and two frequencies (Table 2).

APD modules can be added for short-distance signal acquisitions. APD modules do not affect other channels because they do not emit light, adding APD modules can therefore be performed by only setting their frequencies and timings corresponding to light signals they should detect. The multidistance measurement with a high-density probe arrangement thus can be performed by just adding and setting APD modules.

### 3.6 Light-Emitting Diode Module

**Fig. 10:**
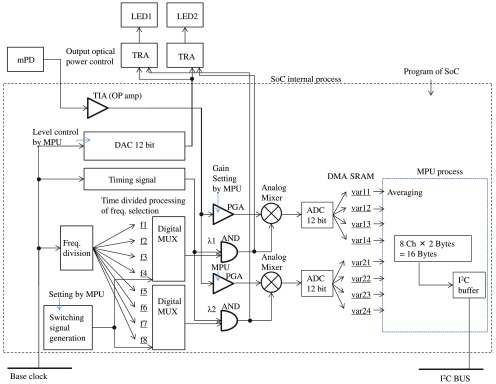
Block diagram for LED module.

A block diagram of an LED module is shown in Fig. 10. Frequency for amplitude modulation is generated by dividing the BC. The optical output is controlled by digital-to-analog converters for power level control and transistors (TRA) for switching. Output level, timing signal, and frequency selection are controlled by parameters stored in an $I^{2}C$ buffer in an SoC static random-access memory (SRAM). Frequency is selected by digital multiplexers (MUX). The monitor photodiode (mPD) output is amplified at a TIA and PGA, the gain of which can be set by an MPU. Detected signals are analog-to-digital converted for each wavelength λ and stored at the BC rate in each memory field automatically by a MPU process. The data are averaged among specific numbers and stored as power output data, which can be used for APC.

### 3.7 Avalanche Photodiode Module

**Fig. 11:**
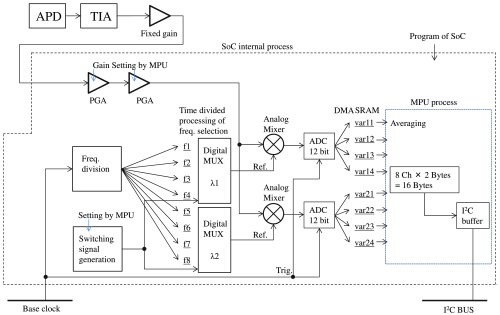
Block diagram for APD module.

A block diagram of an APD module is shown in Fig. 11. Four-channel data are simultaneously obtained the most by dividing detection times (1–4). An APD is used for a detector and the photocurrent signal is amplified at a TIA and one fixed amplifier and two PGAs. Six or more frequencies are generated by dividing the BC, and any frequency can be technically set for each time division at each wavelength λ (f1−f8). Frequency is selected at each detection timing by digital MUX. The reference signal of modulation frequencies and optical signal amplified by PGAs is multiplied at an analog mixer in a hardware lock-in process. The processed signals are A/D converted at the BC, and all digitalized data are stored in SRAM via dynamic memory access in the SoC. The MPU processes averaging the data and the lock-in signal is obtained at each timing and each wavelength λ. The averaged data are recorded in the $I^{2}C$ buffer and accessed (acquired) by the control unit via the $I^{2}C$ bus. A high voltage supplier (HV) for the APD is also implemented in the module. For high voltage control, 3.3-V supplied voltage is boosted from 80 to 90-V reverse voltage by a DC-to-DC converter.

### 3.8 Automatic Power and Gain Control

The LED module has an APC function. This is an MPU-processed function of monitoring the power of an LED and making it constant by controlling the driving current for the LED. The signals of two wavelengths are separately detected by analog lock-in detection because two wavelengths of light are simultaneously detected by a single mPD. The driving currents for two wavelengths can be independently controlled. A proportional-integral-derivative (PID) controller is implemented in MPU processing, and the parameters are stored in the internal memory. The optical output power can be changed on a PC through the control unit.

**Fig. 12:**
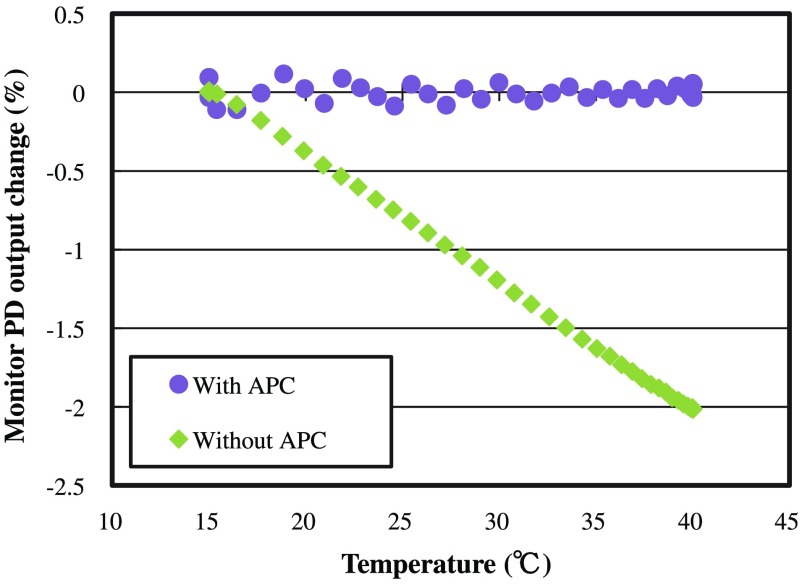
Output power change of LED depending on temperature when APC with PID is used or not.

A test was conducted to demonstrate the performance of APC. The temperature of the LED module was changed by Peltier controller, and power output of mPD was monitored. Output power change (mPD based) of the LED depending on temperature when APC with PID was used or not is shown in Fig. 12. The output power change of the LED was controlled within 0.1% (standard deviation: 0.05%).

The APD modules also perform AGC of optical detection. Each APD module sets each gain of PGA to obtain the suitable signal amplitude. Such autonomous AGC can be simultaneously performed at all channels because each module has an MPU in an SoC.

### 3.9 Fitting Indicator of Headset

Because each LED/APD module has an MPU in an SoC that autonomously controls optical power and detector gain, each module has information of the fitting status of a headset (holder of modules placed on the head). Using this advantage, the module has a color LED indicator on the module case so that the color of the LED indicator tells users the fitting status of the headset, which contributes to shortening the preparation time of the measurement. No additional electric wire is necessary between each module and control unit.

## 4 Human Brain Measurement

To evaluate the developed system, human brain activity in the left prefrontal area of an adult male participant during a verbal fluency task^11^ was measured. The purpose of this measurement is not to know human brain activity responding to a specific task but to check Hb signals obtained by the system.

The verbal fluency task consists of a 60-s task period, and pre- and post-task control periods (20 to 30 s for each). During the task period, a participant was requested to verbalize as many words as possible beginning with a specific Japanese character (randomly presented). In the control period, the participant was requested to verbalize five Japanese vowels repeatedly.

**Fig. 13:**
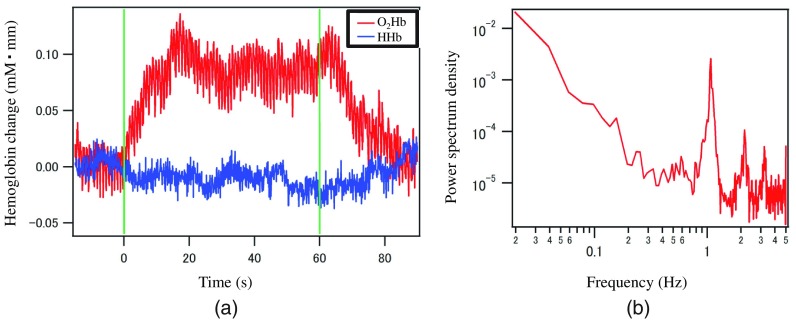
Observed signals of Hb change in human. (a) Hb changes during verbal fluency task for single trial with HPF (0.008 Hz). (b) Power spectrum density of $O_{2}\mathrm{Hb}$ signal.

Hb signals were obtained in accordance with the standards of the internal review board on Research & Development group, Hitachi, Ltd. The measured oxygenated- and deoxygenated-Hb signals ($O_{2}\mathrm{Hb}$ and HHb) after applying a 0.008-Hz high-pass filter (HPF) and power spectrum density during a one-trial verbal fluency task for $O_{2}\mathrm{Hb}$ are shown in Fig. 13. Increased $O_{2}\mathrm{Hb}$ signals by around 0.1  mM·mm during a task period was obtained without averaging. A heart rate around 1 Hz was clearly seen in the power spectrum density.

## 5 Conclusion

We developed a freely rearrangeable and exchangeable optical module with an SoC for a wearable fNIRS system. By decreasing analog circuits using the SoC, low cost and small LED and APD modules have been developed that can be used for normal and high-density arrangements. The new concept of a module-based fNIRS device enables flexibility in probe arrangements and source–detector combination settings and even enables modules to be easily replaced and added. These modules can be used for measurements from one-channel to multichannel brain-activity monitoring, broadening the applicability of the wearable fNIRS system.
